# Time-varying analysis of electrodermal activity during exercise

**DOI:** 10.1371/journal.pone.0198328

**Published:** 2018-06-01

**Authors:** Hugo F. Posada-Quintero, Natasa Reljin, Craig Mills, Ian Mills, John P. Florian, Jaci L. VanHeest, Ki H. Chon

**Affiliations:** 1 University of Connecticut, Storrs, CT, United States of America; 2 Navy Experimental Diving Unit, Panama City, FL, United States of America; Universita degli Studi di Pisa, ITALY

## Abstract

The electrodermal activity (EDA) is a useful tool for assessing skin sympathetic nervous activity. Using spectral analysis of EDA data at rest, we have previously found that the spectral band which is the most sensitive to central sympathetic control is largely confined to 0.045 to 0.25 Hz. However, the frequency band associated with sympathetic control in EDA has not been studied for exercise conditions. Establishing the band limits more precisely is important to ensure the accuracy and sensitivity of the technique. As exercise intensity increases, it is intuitive that the frequencies associated with the autonomic dynamics should also increase accordingly. Hence, the aim of this study was to examine the appropriate frequency band associated with the sympathetic nervous system in the EDA signal during exercise. Eighteen healthy subjects underwent a sub-maximal exercise test, including a resting period, walking, and running, until achieving 85% of maximum heart rate. Both EDA and ECG data were measured simultaneously for all subjects. The ECG was used to monitor subjects’ instantaneous heart rate, which was used to set the experiment’s end point. We found that the upper bound of the frequency band (Fmax) containing the EDA spectral power significantly shifted to higher frequencies when subjects underwent prolonged low-intensity (Fmax ~ 0.28) and vigorous-intensity exercise (Fmax ~ 0.37 Hz) when compared to the resting condition. In summary, we have found shifting of the sympathetic dynamics to higher frequencies in the EDA signal when subjects undergo physical activity.

## Introduction

Electrodermal activity (EDA) has recently garnered interest as an alternative for assessing sympathetic dynamics because sweat glands are only innervated by sympathetic nerves [1]. Eccrine sweat glands (which are in the cholinergic part of the sympathetic system) were initially thought to respond only to peripheral stimuli—as during thermoregulatory sweating. However, in response to pharmacological central depressants, the electrodermal response is inhibited in a manner analogous to other sympathetic systems [2,3]. A central adrenergic inhibitory mechanism may also be involved [3,4]. For this reason, even though EDA may be modified peripherally, it has been suggested as a model to study central sympathetic activation [5,6].

EDA is technically defined as a measure of the changes in electrical conductance of the skin. EDA, as a reflection of autonomic innervation of sweat glands [7], is thought to provide a quantitative functional measure of sudomotor activity [8,9]. Some studies have used EDA to assess sympathetic function during exercise [10,11]. When subjects undergo physical load, EDA is increased as sweating rate increases, as a product of an initial recruitment of sweat glands and then an increased sweat secretion per gland [12,13]. Although there is an evident increase in the level of EDA, most valuable information from EDA resides not only in the skin conductance level, but also in the oscillatory patterns [14].

In the time domain, typically two measures are obtained from the EDA: the skin conductance level (SCL) and the non-specific skin conductance responses (NS.SCRs) [15]. SCL (microsiemens, μS) is a measure related to the slow shifts of EDA, and is computed as a mean of several measurements taken during a specific period. The skin conductance responses (SCRs) are the rapid transient events contained in the EDA signals. The NS.SCRs index is computed as the number of SCRs in a period of time.

Recently, we have used power spectral density (PSD) and time-varying spectral analysis of EDA to obtain information about the spectral distribution of sympathetic arousal in the skin. We observed that the dynamics of the sympathetic system estimated from the EDA signal under non-exercise conditions were mainly confined to the low-frequency range, from 0.045 to 0.25 Hz, when subjects underwent stress-invoking tests like cold pressor test, Stroop task, and standing test [16,17]. Using heart rate variability (HRV), several studies have shown the increase in frequencies of autonomic control during exercise [18–21]. Although central and skin sympathetic control systems are obviously different, the increase in frequencies of central sympathetic control suggests that the same phenomenon could be noticeable at the skin level.

We hypothesize that the frequency bandwidth of the sympathetic dynamics of EDA is modified under physical activity. To date, we are not aware of any reported studies exploring changes in the frequency dynamics of EDA during exercise. Hence, to test whether or not the frequency band shifts to higher frequencies, we conducted submaximal physical tests on a treadmill with collection of both electrocardiogram (ECG) and EDA signals from healthy subjects.

## Materials and methods

### Protocol

All procedures performed in studies involving human participants were in accordance with the ethical standards of the institutional and/or national research committee and with the 1964 Helsinki declaration and its later amendments or comparable ethical standards. Informed written consent was obtained from all individual participants included in the study. This protocol was approved by the Institutional Review Board of the University of Connecticut.

Subjects for whom exercise represents a low risk level, based on standardized guidelines from the American College of Sports Medicine (ACSM) [22], were asked to participate in the study. Participants were recruited between May and October 2016. An advertising poster was attached to bulletin boards throughout Storrs campus of the University of Connecticut to draw attention from prospective participants. After arranging a meeting with them via phone or e-mail, researchers performing the experiment discussed the study with the prospective participants. Eighteen healthy subjects, 11 males and 7 females, aged 21 ± 3 years, were enrolled. None of the participants dropped-out the experiment. Participants were asked to avoid caffeine and alcohol during the 48 hours preceding the test, and were instructed to fast (water only) for at least 3 hours before testing. The study was conducted in a quiet, comfortable room (ambient temperature, 18–20°C, and relative humidity between 30–50%).

Before the exercise test began (i.e. prior to the protocol described in Table 1), the subjects were asked to lay in the supine position for 5 minutes to procure hemodynamic stabilization prior to 5 minutes of data collection in this position. ECG and EDA were measured simultaneously for each subject throughout the entire experiment. The ECG signal, from an HP ECG monitor (HP 78354A), was used to monitor the subject’s HR. For EDA, a galvanic skin response (GSR) module from ADInstruments was used. Three hydrogel silver-silver choloride electrodes were used for ECG signal collection. The electrodes were placed on the shoulders and lower left rib. In addition, a pair of stainless steel electrodes were placed on index and middle fingers of the right hand to collect the EDA signal. Subjects were instructed to keep their right hand stable, raised at chest height. The skin was cleaned with alcohol before placing the ECG and EDA electrodes. The leads were taped to the subject’s skin using latex-free tape, to avoid movement of the cables, which can corrupt the signals. All signals were acquired through the ADInstruments analog-to-digital converter, and compatible PowerLab software, while the sampling frequency was fixed to 400 Hz for all signals. Participants were asked to wear their own active wear/gym clothes during the protocol with the shirt covering the electrodes and cables during the experiment.

**Table 1:** Experimental protocol.

Stage	Action	Speed	Duration
1	Rest (supine)		5 min
2	Stand, move to treadmill		2 min
3	Warm up by walking	3 mi/h (~4.82km/h)	3 min
4	Walk	3 mi/h (~4.82km/h)	2 min
5	Start running	5 mi/h (~8 km/h)	2 min
6	Accelerate running until reach 85% of HRmax	+ 0.6 mi/h/min (+ ~1km/h/min)	>2 min
	Run slower (recovery starts)	5 mi/h (~8 km/h)	4 min
	Walk (recover)	3 mi/h (~4.82km/h)	5 min
	Rest (supine)		10 min

The experimental protocol resembled the procedure used previously [23] (Table 1). Notice that this study involves only sub-maximal intensity physical activity, 85% of a subject’s maximum heart rate (HRmax). Subjects were first monitored for 5 minutes at rest (supine, without any movement or talking) to measure resting HR and EDA. The subjects then performed the incremental test on a motorized treadmill (Life Fitness F3). 85% HRmax was calculated from the equation [22]:

$${HR}_{max}=206.9-(0.67*age)$$ (1)

The incremental running began with an initial warm-up, followed by walking at 3 mi/h (~ 4.82 km/h). The speed was increased to 5 mi/h (~ 8 km/h) and increased 0.6 mi/h (about 1 km/h) every subsequent minute until the subjects reached 85% of their HRmax. When a subject reached 85% of HRmax within 2 minutes of running, the data were excluded because at least 2 minutes of data were required for processing. The 18 subjects enrolled for this study represent those who were able to provide at least 2 minutes of data prior to reaching 85% of HRmax. After subjects reached 85% of their HRmax, the treadmill speed was reduced to 5 mi/h (~ 8 km/h) for another 4 minutes to start the recovery phase, followed by walking at 3 mi/h (about 4.82 km/h) for 5 minutes. A final 10 minute period (or more if needed to achieve baseline HR) in the supine position was used to allow HR to return to baseline. The duration of the experiment was approximately one hour.

### Signal processing

We define six stages of exercise in the data collection: (a) stage 1—the subject is lying down on their back, (b) stage 2—the subject stands and moves to the treadmill, (c) stage 3—the subject warms up by walking on the treadmill, (d) stage 4—the subject walks on the treadmill at 3 mi/h, (e) stage 5—the speed is set to 5 mi/h, and (f) stage 6—increasing treadmill speed until the subject is running at 85%-HRmax (see Table 1). Although stages 3 and 4 are in the low-intensity range of exercise, stage 4 is a higher exercise intensity compared to stage 3, as the accumulated time of exercise contributes to increment the intensity. Two minute segments of data from every stage, including running at 85%-HRmax (stage 6), were used for data analysis. Using these various exercise stage signals, we tested how the spectral content of EDA evolves throughout the exercise protocol using both time-invariant and time-varying approaches.

### Maximum frequency, Fmax

In order to study the changes in frequency distribution of EDA during exercise, we have defined the maximum frequency, termed Fmax, as the upper frequency bound of the sympathetic components of EDA. The criterion to define Fmax is to find a frequency such that the integration of the spectral power from it to Fs/2 (half of the sampling frequency, or the Nyquist frequency) consists of less than 5% of the total power. For the time-varying approach [24], instantaneous Fmax was computed for every time point, and averaged over the two-minute period. Recently, using equivalent criteria, our time-invariant and time-varying analyses found that in the non-exercise conditions Fmax was 0.25 Hz. Likewise, in this study, we analyzed Fmax for all subjects, to test the effects of physical activity on the possible increase of spectral power for frequencies beyond 0.25 Hz.

### Spectral analyses

Time-invariant and time-varying spectral analyses have been deployed recently on EDA data [16,17]. For this study, EDA data were down sampled to 4 Hz prior to spectral analysis. The time-invariant power spectra of EDA signals were calculated using Welch’s periodogram method with 50% data overlap. A Blackman window (length of 128 points) was applied to each segment, the Fast Fourier Transform was calculated for each windowed segment, and the power spectra of the segments were averaged.

To compute the time-frequency representation (TFR) of EDA we employed variable frequency complex demodulation, a time-frequency spectral analysis technique that provides accurate amplitude estimates and one of the highest time-frequency resolutions. The technique has been previously described [24,25]. We used the instantaneous spectral estimate for every time point of the TFR to compute an Fmax series. We averaged the Fmax series over the time segments defined previously for the six stages. To compute SCL and NS.SRCs, the EDA data were decomposed into tonic and phasic components using the convex optimization approach [26].

### Statistics

Repeated measurements analysis was deployed to evaluate the significance of the effect of increased level of exercise on Fmax. The normality of Fmax in the six stages was tested using the one-sample Kolmogorov-Smirnov test [27–29]. As Fmax was found normally-distributed in both approaches (time-invariant and time-varying), the one-way analysis of variance (ANOVA) was performed to test for significant differences between stages. The Bonferroni method was used for correction of multiple comparisons.

## Results

This section comprises the results of this study, including a general description of the signals obtained during the experiment, their main characteristics, the parameters computed using spectral analysis, and the statistical analysis carried out to evaluate the significance of the results.

**Fig 1:**
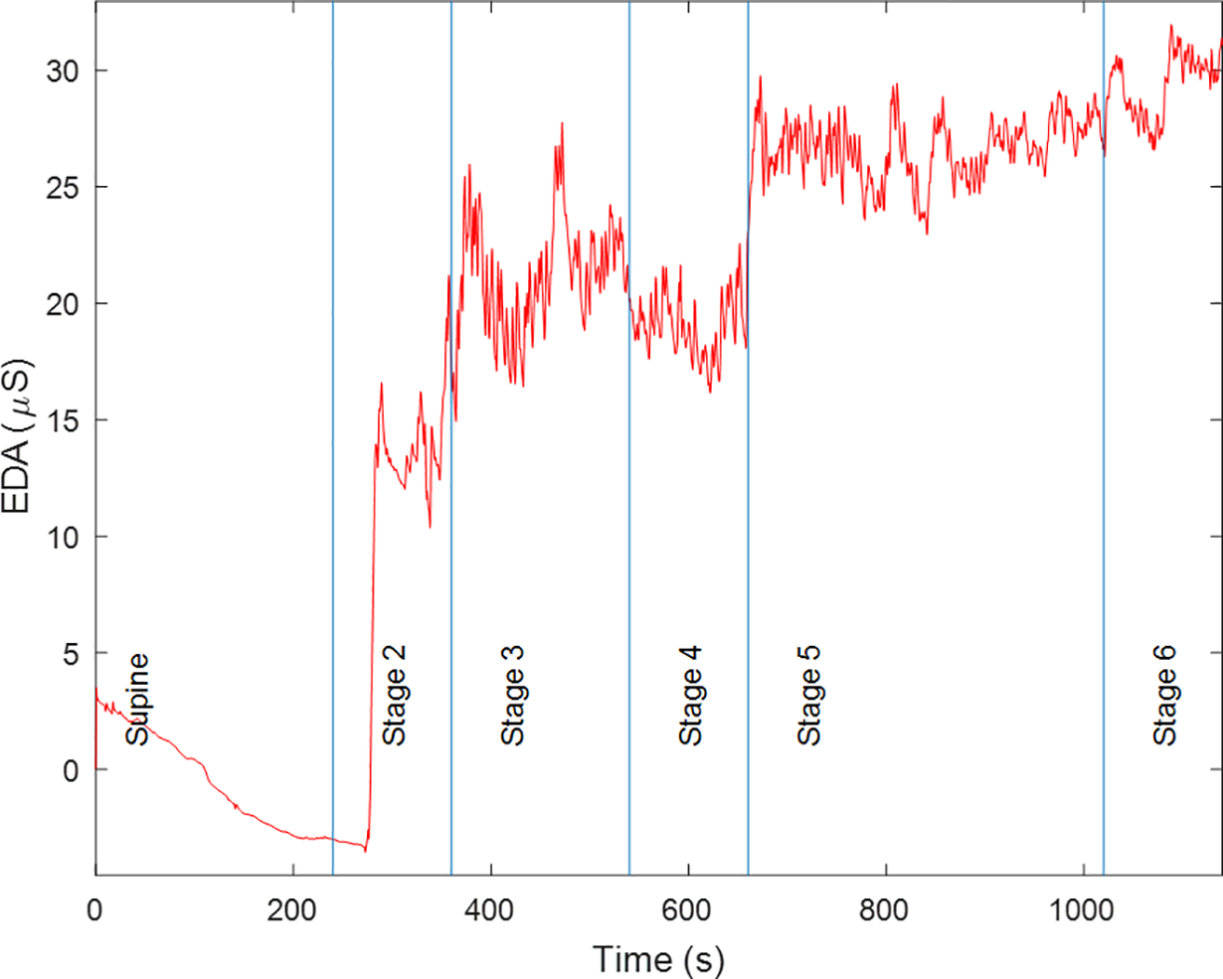
Raw EDA signal for a given subject throughout different body positions and exercise stages.

Fig 1 shows the EDA signal for a representative subject, showing the time progression from lying supine until the moment their HR reached 85% of HRmax. Subjects took 4 minutes and 45 seconds, on average, to reach 85% of HRmax (stage 6). The EDA signal dynamics, shown in Fig 1 for a representative subject, were found to be similar for all subjects. Changes in the conduction levels of EDA are seen with the progression of the various exercise stages. For example, for this subject in the supine posture, the SCL is low, followed by a marked increase in conductance when the subject stood up to go to the treadmill. There is also a considerable increase in SCL when the subject started walking (stage 3). The mean conductance level tended to increase during exercise (stages 3, 4, 5, and 6). Lines in Fig 1 denote the time when the subject started in the supine position followed by performing various stages of exercise.

In addition to the increase in conductance level, there is also an increase in high-frequency components, as shown in Fig 1. A good indicator of the quality of the EDA signal is the smoothness of the raw waveform [15]. Since the SCRs that we obtained in this experiment are not due to any specific instantaneous stimuli, in contrast to startle-like experiments, they are NS.SCRs. Note that the occurrence of NS.SCRs is more frequent when the intensity of the exercise is increased. Although NS.SCRs have been traditionally quantified in the time domain, by counting them and providing an index of number of NS.SCRs per unit of time, these high-frequency oscillations have shown to be an even more sensitive index of sympathetic arousal when analyzed in the frequency or time-frequency domains [16,17]. The SCL, NS.SCRs, and % HRmax values are included in Table 2, along with the p values obtained from the test for significant differences among stages. As expected, the % HRmax value increased with the exercise intensity [30]. The SCL was significantly different in stages 3 to 6 compared to stages 1 and 2, and between stage 6 and 3. The NS.SCRs index was also significantly different in stages 3 to 6 compared to stages 1 and 2, and between stages 2 and 1.

**Table 2:** Fmax, EDA measures and % HRmax for different stages.

	Fmax (Hz)		EDA measures		
Stage	Time-invariant analysis	Time-varying analysis	SCL (μS)	NS.SCRs (count/min)	% HRmax
**1**	0.14 ± 0.085	0.11 ± 0.075	-0.032 ± 3.27	20.5 ± 5.99	34.2 ± 6.93
**2**	0.16 ± 0.12	0.14 ± 0.081	1.19 ± 3.77	25 ± 2.51	43.7 ± 7.72
**3**	0.22 ± 0.12	0.23 ± 0.077	8.3 ± 6.26	29.4 ± 3.72	51.1 ± 7.98
**4**	0.28 ± 0.091	0.27 ± 0.067	11 ± 7.51	30.4 ± 2.89	52.5 ± 9.5
**5**	0.27 ± 0.16	0.31 ± 0.12	13.7 ± 8.2	31.3 ± 2.58	69.6 ± 9.77
**6**	0.31 ± 0.15	0.37 ± 0.11	15.1 ± 9.24	31.5 ± 2.52	81.8 ± 6.39
**p-value**	7.30E-05	2.40E-14	1.20E-11	2.70E-17	3.9E-34

Values are mean ± standard deviation

*p-value* for the null hypothesis that the means of the values in the different stages are equal.

*SCL* skin conductance level, *NS*.*SCRs* non-specific skin conductance responses, *HRmax* maximum heart rate

**Fig 2:**
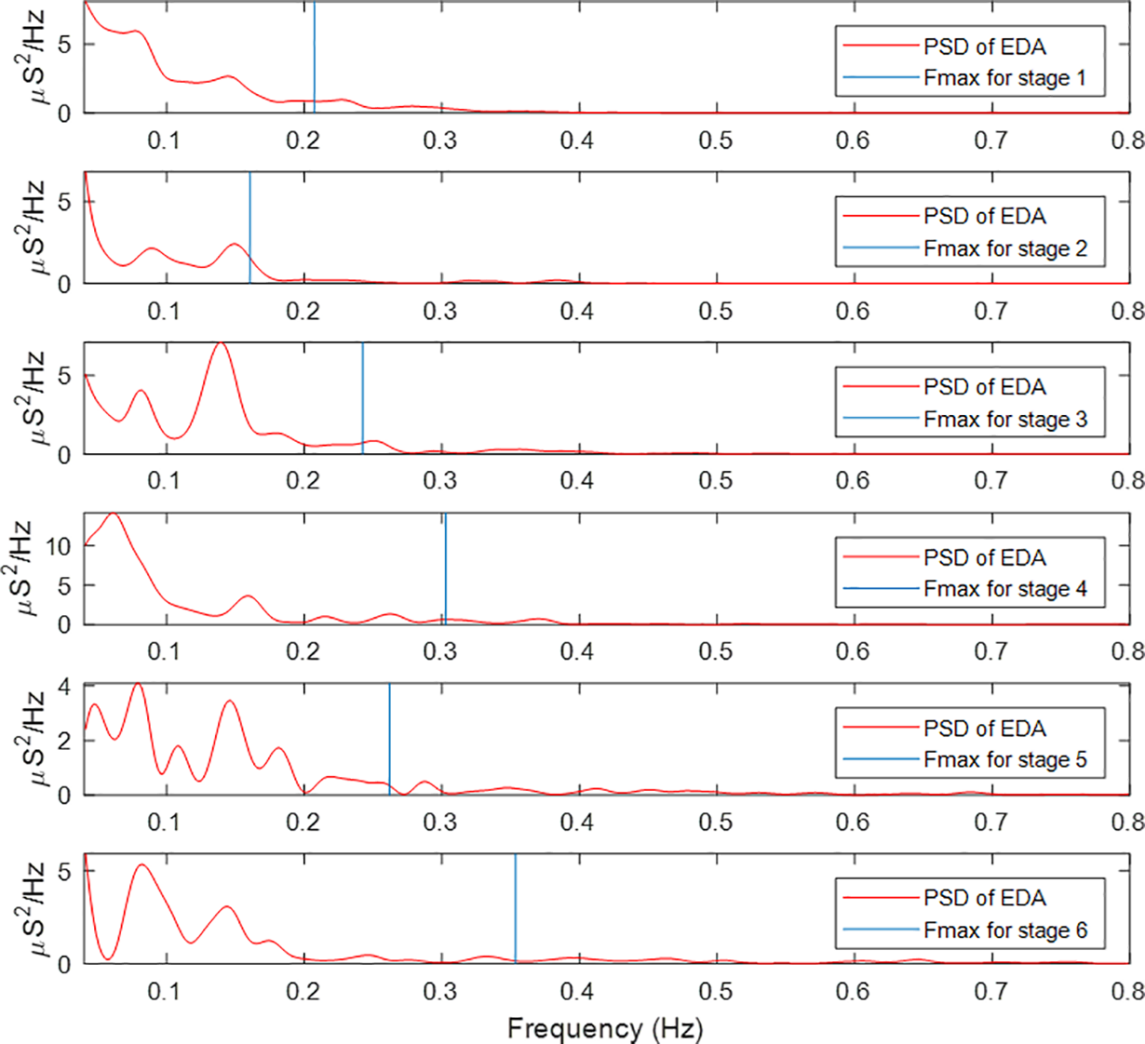
Power spectral density of EDA for a given subject during different events and computed Fmax. Fmax values increase concomitantly with exercise intensity.

Power spectra of EDA signal segments were computed for all subjects and for every stage of exercise. Fig 2 includes representative results for one subject. For each spectrum, Fmax was computed and is noted as the vertical blue line. For this representative subject, when the subject was in the supine posture, most of the spectral power was concentrated in the very-low frequency range, although Fmax was located around 0.2 Hz. As the subject stood up (the second panel: stage 2), Fmax was about 0.15 Hz. After the subject had walked for about 5 minutes (stage 4), spectral power shifted towards higher frequencies, reaching an Fmax value of about 0.3 Hz. Fmax exhibited its highest value during stage 6, around 0.35 Hz.

**Fig 3:**
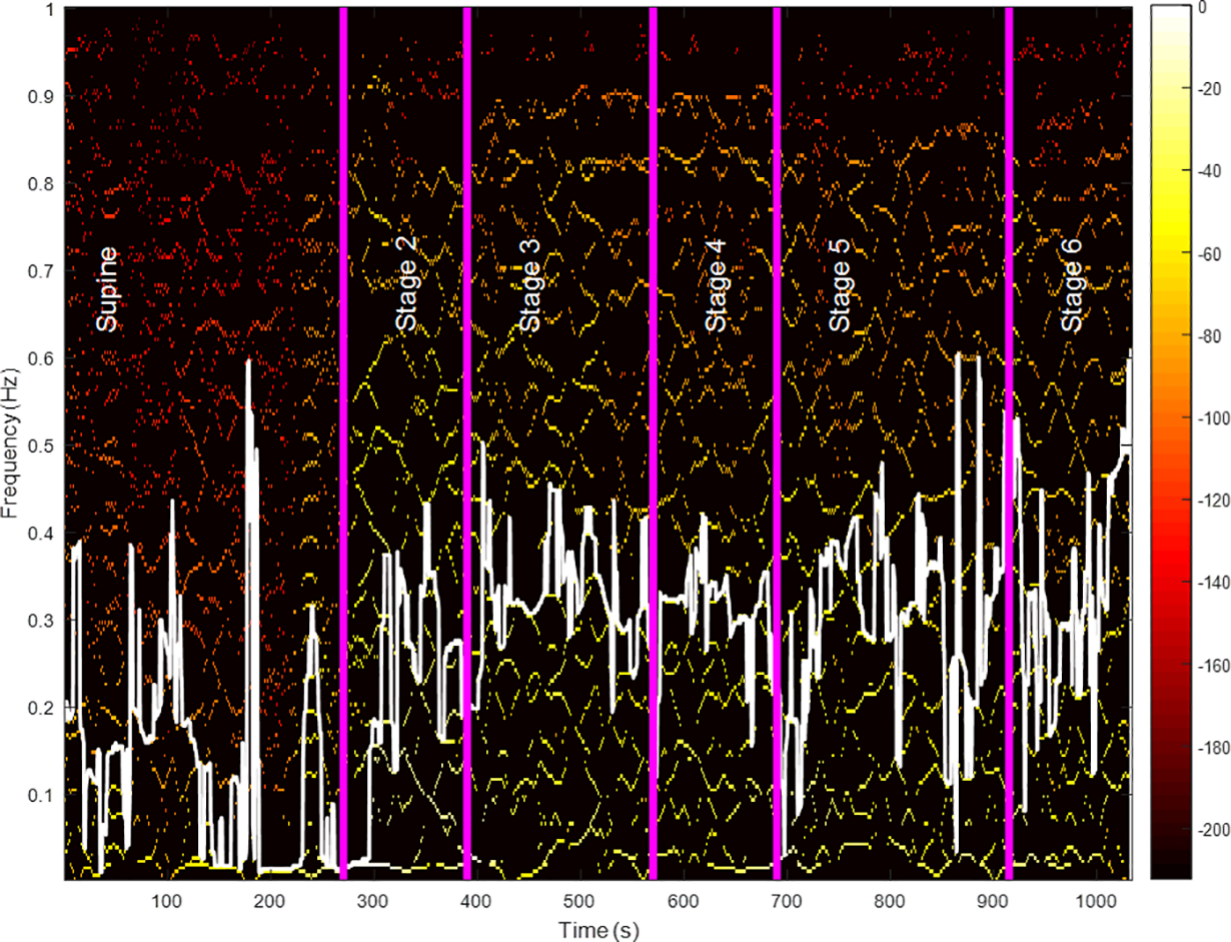
Time-frequency representation of EDA for a given subject. Vertical lines demarcate the transition between stages. Instantaneous Fmax (white line) is computed for each time point.

**Fig 4:**
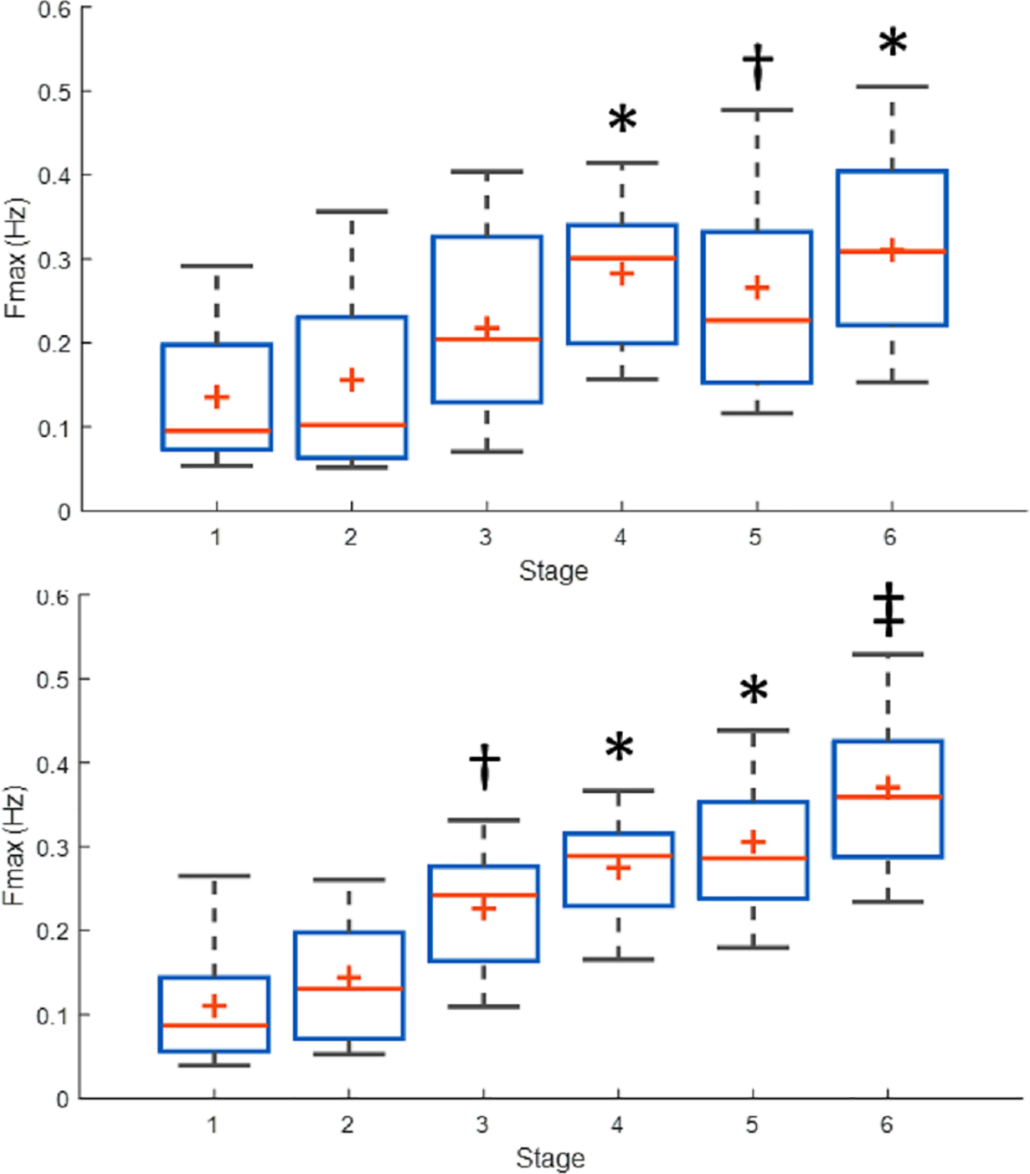
Box plots for estimated Fmax for all exercise stages and subjects. Time-invariant (top panel) and time-varying (bottom panel) approaches. In each box, the central mark is the median, and the edges of the box are the 25th and 75th percentiles. (*) represents significant difference to stages 1 and 2. (†) represents significant difference to stage 1. (‡) represents significant difference to stages 1 through 4.

Fig 3 shows an example of how higher-frequency power was increased over time. Yellow lines are more visible as the subject transitioned to higher stages, and Fmax (shown as a white line in Fig 3) moved upward. Table 2 includes the results for Fmax for both time-invariant and time-varying analyses. Both approaches showed an increase of Fmax for higher exercise intensities. Fmax was higher during stage 5 (running) when compared to stage 1 (supine). Fig 4 illustrates the box plot for the estimates of Fmax using time-invariant and time-varying approaches. The mean value of Fmax was stable for the first two stages, then increased to a medium level when subjects started walking, followed by an increase to a value between 0.31 to 0.37 Hz when subjects underwent higher-intensity exercise.

Using repeated measurements analysis, we found a significant effect of increased exercise intensity in Fmax, for both time-invariant and time-varying approaches. Fig 4 also shows the outcome of multiple comparisons using ANOVA. These results suggest an increase in Fmax at higher intensities of exercise, beginning when the subjects started walking (stage 3), until the subjects were running at 85% HRmax (stage 6). No statistical differences were found between stages 1, 2 and 3 using the time-invariant approach. That approach did, however, show differences in Fmax between the time the subjects were exercising in stages 4, 5 and 6, compared to stage 1. Fmax was also different between stages 4 and 6, compared to stage 2. As mentioned, the time-invariant PSD approach estimated that the mean Fmax during stage 5 was non-significantly different than during stages 3 or 4.

Using the time-varying approach, we found differences between stage 3 and stage 1. In other words, Fmax was significantly increased when the subject walked, compared to their being in the supine position. Furthermore, this approach found differences more consistently for the three highest levels of physical activity, compared to the first two stages. This means that Fmax was significantly higher at the time the subjects had walked for about 5 minutes (stage 4) and at any level of running (stages 5 and 6), compared to the two first stages (supine and standing). Also, Fmax at stage 6 (running at 85% HRmax) was found to be significantly higher compared to stages 3 and 4, which suggests that at higher exercise intensity, the spectral components of EDA shift to higher frequencies, compared to low-intensity exercise (walking).

## Discussion

We have found evidence of an increase in Fmax, the maximum frequency of sympathetic control on the EDA signal, concomitant with exercise intensity. The results are based on both time-invariant and time-varying spectral analyses of EDA; albeit more significant shifts were found with the latter approach. It implies that using a fixed frequency band for rest and all exercise intensities can lead to inaccurate capture of the sympathetic dynamics. As shown in Fig 2, we found that the EDA sympathetic dynamics’ upper frequency band shifts to higher frequencies with increasing exercise intensities.

Oscillations of the EDA signals have been previously investigated [14,16,17]. Besides the known transient in the skin conductance level, low-frequency oscillatory patterns were first observed in the EDA signal under physical load (hand grip), mental load and alarm reaction [14]. In recent studies, we found an increase in the power of EDA in the range of 0.045 to 0.25 Hz under cognitive, orthostatic and physical stress [16,17]. For the set of subjects undergoing the above-noted variety of stress tests, the 0.25 Hz bound accounted for 95% of the spectral power in the analysis. However, these experiments did not provide information on how the spectral distribution of EDA may change under physical exercise. In the present study, we collected EDA data during an increasing exercise intensity test to elucidate the dynamics of the spectra of the skin-level sympathetic control.

EDA exhibited spectral components of maximum frequency around an average of 0.1 Hz, during supine and standing up stages (Table 2). This frequency is in agreement with the oscillatory patterns observed in a study conducted years ago [14]. The sympathetic modulation’s mean upper frequency bound shifted higher to around 0.15 Hz when the subjects transitioned from supine to upright posture and began walking (stages 1, 2, and 3, respectively) (Table 2). This shift was significant in the time-varying approach. Stage 3 corresponds to low-intensity exercise, during which the contribution of the ANS is thought to be a combination of withdrawal of the parasympathetic tone, and a small increase of sympathetic tone. In our previous studies, we found that sympathetic control was confined to the range 0.045 to 0.25 Hz, when subjects underwent the cold pressor test, tilt table test, cognitive stress and orthostatic stress [16,17]. During stage 3, subjects exhibited similar sympathetic tone as seen with the various stressors used in our prior studies.

Stages 1 to 4 consist of low-intensity levels of exercise. Nevertheless, each stage represents an increase in exercise intensity. For example, stage 4 (walking for 3+ minutes) represents a slightly higher exercise intensity compared to stage 3 (start walking), as the duration of the exercise contributes to increased intensity. % HRmax (Table 2) also shows a higher exercise intensity, as the HR is an indication of the intensity of the exercise [30]. The last three stages (transition from low to vigorous-intensity exercise) are characterized by the increase in sympathetic tone. In terms of spectral distribution, we found that the sympathetic modulation in the EDA signal moved to frequencies beyond 0.25 Hz. When subjects underwent stage 4, the increase of Fmax was found to be significant with respect to stages 1 and 2, in both approaches. The mean upper frequency bound during this stage was located around 0.28 Hz. When the subjects commenced stage 5 (running), we observe that the mean Fmax shifted to about 0.3 Hz, although such increase was not significant with respect to stage 4. However, the mean Fmax increased to about 0.37 Hz when subjects were at stage 6 (running at a pace associated with 85% HRmax), being significantly higher than stages 1 through 4, in the time-varying approach. Notice that this shift in frequencies in the EDA signal introduced by physical activity was never observed to be produced by other types of stress.

The time-domain measures of EDA (e.g. SCL and NS.SCRs) are commonly utilized as markers of sympathetic arousal in response to tonic stimuli [15]. However, computing NS.SCRs relies on either manual or automatic SCR detection, which is usually more cumbersome and time consuming. Furthermore, these measures are highly variable and less sensitive than indices based on frequency or time-frequency analysis of EDA [16,16,17,31]. In this study we found that SCL and NS.SCRs significantly increased with the increase of exercise intensity. In general, the indices exhibited significant differences between higher exercise intensities (stages 3 through 6) and lower exercise intensities (stages 1 and 2). However, time-varying spectral analysis is more sensitive than time-domain analysis, as in addition to the significant differences noted above, we also observe a difference in Fmax between stage 6 and stages 1 to 4.

Although 85% of the standard HRmax parameter is widely accepted as a safe end-point for submaximal exercise tests [22,23], and was suitable for the purposes of this study as it allowed us to observe the increase in heart rate and spectral components of EDA, using standardized HRmax may not be the optimal choice for identifying high intensity physical activity. This is because how we obtain HRmax only factors in age, but other factors such as gender, regularity of physical activity, genetics, habits, weight, and so forth can influence HRmax. We had to use 85% of standard HRmax to minimize the risk to subjects, but certainly high-intensity physical activity may require a different threshold for HRmax as well as a different means to obtain HRmax.

Other limitation of the study is the indirect measurement of the sympathetic dynamics via EDA. More direct measures of the sympathetic dynamics, such as the use of microneurography, would have been beneficial to validate the EDA measurements. Moreover, the use of drugs that block the autonomic nervous system, such as atropine, during exercise will help to further validate the suggested frequency bands via the EDA. These experiments are planned for further investigations. Furthermore, this study was carried out in a controlled experimental setup. In a real-life scenario, motion artifacts could become a potential source of signal corruption. The reliability of EDA can also be diminished by excessive sweating.

### Perspectives and significance

Spectral analysis of EDA can lead to a better understanding of the effect of physical exercise on the human autonomic nervous system. EDA could be extended to other studies to better characterize and discriminate autonomic nervous system dynamics that may be indicative of fatigue, stress or exercise intolerance. An accurate understanding of the autonomic nervous system’s dynamics under exercise stress can lead to better guidance and interventions to improve human health and performance.

## Conclusion

Previous studies have shown that under resting conditions most of the sympathetic spectral power of EDA is confined to the range 0.045 to 0.25 Hz. In this study, we have found that when subjects undergo higher exercise intensities, the upper band of the sympathetic dynamics shifts to 0.28 to 0.37 Hz. The varying upper frequency band of the sympathetic dynamics should be considered to provide a better understanding of the functioning of sympathetic control, especially during higher-intensity physical activity. We conclude, similar to previous studies [14], that assessment of EDA may be a useful tool in the evaluation of the interaction between different autonomic regulatory processes that are carried out by the common brainstem system.

## Abbreviations

ANS: autonomic nervous system
ECG: electrocardiogram
EDA: electrodermal activity
Fmax: upper frequency bound of the sympathetic components of EDA
HR: heart rate
HRmax: maximum heart rate
NS.SCRs: non-specific skin conductance responses
PSD: power spectral density
SCL: skin conductance level
TFR: : time-frequency representation
VFCDM: variable frequency complex demodulation
